# Stratification of responders towards eculizumab using a structural epitope mapping strategy

**DOI:** 10.1038/srep31365

**Published:** 2016-08-11

**Authors:** Anna-Luisa Volk, Francis Jingxin Hu, Magnus M. Berglund, Erik Nordling, Patrik Strömberg, Mathias Uhlen, Johan Rockberg

**Affiliations:** 1 KTH - Royal Institute of Technology, School of Biotechnology, Department of Proteomics and Nanobiotechnology, 106 91 Stockholm, Sweden; 2 KTH Center for Applied Proteomics, School of Biotechnology, KTH - Royal Institute of Technology, Stockholm, Sweden; 3 Swedish Orphan Biovitrum AB, 11276 Stockholm, Sweden; 4 KTH - Royal Institute of Technology, Science for Life Laboratory, 17165 Stockholm, Sweden; 5 Novo Nordisk Foundation Center for Biosustainability, Technical University of Denmark, DK-2970 Hørsholm, Denmark

## Abstract

The complement component 5 (C5)-binding antibody eculizumab is used to treat patients with paroxysmal nocturnal hemoglobinuria (PNH) and atypical haemolytic uremic syndrome (aHUS). As recently reported there is a need for a precise classification of eculizumab responsive patients to allow for a safe and cost-effective treatment. To allow for such stratification, knowledge of the precise binding site of the drug on its target is crucial. Using a structural epitope mapping strategy based on bacterial surface display, flow cytometric sorting and validation via haemolytic activity testing, we identified six residues essential for binding of eculizumab to C5. This epitope co-localizes with the contact area recently identified by crystallography and includes positions in C5 mutated in non-responders. The identified epitope also includes residue W917, which is unique for human C5 and explains the observed lack of cross-reactivity for eculizumab with other primates. We could demonstrate that *Ornithodorus moubata* complement inhibitor (OmCI), in contrast to eculizumab, maintained anti-haemolytic function for mutations in any of the six epitope residues, thus representing a possible alternative treatment for patients non-responsive to eculizumab. The method for stratification of patients described here allows for precision medicine and should be applicable to several other diseases and therapeutics.

The term precision medicine describes the idea of providing effective treatment based on a patient’s molecular make up. Recent advances in molecular diagnostic tools and handling of large data sets allow for the stratification of patients based on e.g. genetic or protein information and make it possible to provide tailored treatment for these sub-groups^1^. Here we describe how epitope information, exemplified by binding of the therapeutic antibody eculizumab on its target complement component 5 (C5), can be used to guide treatment.

The complement system is an important part of the human innate immunity. It fulfils an important role in fighting bacterial infections and homeostasis and provides a link between innate and adaptive immune response^2^. Such powerful functions require a tight regulation to prevent the complement system from attacking the host’s cells. Paroxysmal nocturnal hemoglobinuria (PNH) and atypical haemolytic uremic syndrome (aHUS) are two disorders associated with a malfunctioning complement system. PNH is characterized by blood cells deficient of glycosylphosphatidylinositol (GPI)-anchored proteins, which protect cells from complement attack, caused by an acquired mutation in hematopoietic stem cells^3^,^4^,^5^. This consequently results in intravascular haemolysis, which in turn leads to anaemia and thrombosis^4^,^5^,^6^. Atypical HUS is a renal disease that is characterized by an unregulated activation of the alternative pathway of the complement resulting in thrombosis in kidney capillaries and renal failure^7^,^8^.

The monoclonal antibody eculizumab (Soliris®) is the first FDA-approved therapeutic antibody for treatment of PNH^2^,^4^,^5^,^9^ and aHUS^7^,^10^. Eculizumab is a humanized monoclonal antibody targeting C5. The suggested mode of action is that eculizumab binding to C5 prevents the entry and subsequent cleavage of C5 by C5 convertase^7^,^11^. C5 is a central protein in the complement system cascade that is common to all three pathways. This makes it attractive as a therapeutic target. By inhibiting C5 cleavage the common downstream cascade can be disrupted independent of the activating pathway, i.e. no C5b-9 formation (membrane attack complex, MAC) and generation of the anaphylatoxin C5a occurs^2^,^5^. At the same time, the upstream pathways resulting in generation of C3b are kept intact and thus pathogen clearance via C3b-mediated opsonisation is not impaired^4^,^5^. Besides the approved treatment of PNH and aHUS, eculizumab has also proven effective in some cases for treatment of other conditions, like myasthenia gravis^12^,^13^, neuromyelitis optica^14^,^15^, membranoproliferative glomerulonephritis^16^,^17^, catastrophic antiphospholipid syndrome^18^,^19^,^20^ and prevention of rejection after renal transplants^21^,^22^,^23^.

Despite the proven relevance of the drug, reports on non- or poorly responding patients have been published^24^,^25^,^26^,^27^,^28^. Among them a study on Japanese PNH patients which carried a mutation in the C5 gene, which changes the C5 protein so that it can no longer be bound by eculizumab^24^. This highlights the need for an exact determination of eculizumab’s epitope on C5. Such knowledge would enable the development of genotyping kits for the prediction of responsiveness of a patient to eculizumab treatment.

Different efforts have been undertaken to identify the binding site of eculizumab on C5^11^,^29^,^30^. In an earlier report to the Japanese Pharmaceutical and Food Safety Bureau^29^, eculizumab was reported to have a conformational epitope consisting of three distant amino acid stretches covering residues 822 to 826 (DVFLE), 879 to 883 (KSSKC), and 930 to 933 (VPEG). Later, Zuber *et al.*^7^ only refer to KSSKC as eculizumab’s epitope. In a recent publication^30^ based on transmission electron microscopy (TEM), eculizumab was found to interact with the MG7 domain with R885 and the KSSKC peptide being part of the binding site. While the present study was under review the crystal structure of C5 in complex with the eculizumab Fab fragment was solved^11^. While accurately determining the region of binding, the resolution of the co-crystal structure did not allow an unambiguous determination of intermolecular interactions at residue level.

Here, we used an epitope mapping strategy based on deep mutational scanning of surface-displayed protein domains to explore in detail the structural epitope properties of eculizumab. While high-resolution X-ray crystallography of the antibody complex still represents the gold standard for epitope determination^31^ other epitope mapping methods have been developed, like peptide scanning^32^, alanine scanning^33^ and several combinatorial library techniques^34^ that are applicable to a wider range of proteins and offer higher throughput. The approach presented here is based on the gram-positive bacterium *Staphylococcus carnosus* (*S. carnosus)*^35^,^36^, which proved to be advantageous for display applications due to its high mechanical resistance allowing for cell sorting applications and the lack of extracellular proteolytic activity^37^,^38^. For the first time, staphylococcal display was used for mapping of a structural epitope by displaying randomly mutated protein domains on the surface of *S. carnosus*. The combination of random mutagenesis and surface display of protein domains allows a more thorough analysis of the mutational space under native-like conditions in contrast to other high-throughput mapping techniques like peptide arrays and alanine scanning.

The presented workflow (Fig. 1a) includes the identification of a binding domain, creation of a mutant display library, identification and sorting of surface expressed non-binding domain mutants using flow cytometry and sequencing of them. To confirm the identified positions the mutants were produced as full-length proteins in mammalian cells and functionally analysed for haemolytic activity and binding to the antibody. The identified C5 residues involved in the binding to eculizumab were matched against reported germline variations of relevance for the prediction of responders to treatment with this therapeutic antibody, which constitutes the stratification part of the workflow (Fig. 1b). Comparing the patient’s gene sequence with the epitope of available drugs enables stratification into treatment groups. Our work outlines a general strategy for stratification of responders and non-responders for treatment with therapeutic antibodies and more specifically analyses the underlying molecular mechanism for patients’ response to the therapeutic antibody eculizumab.

## Results

### Eculizumab binds to MG7 domain displayed on *Staphylococci*

**Figure 1:**
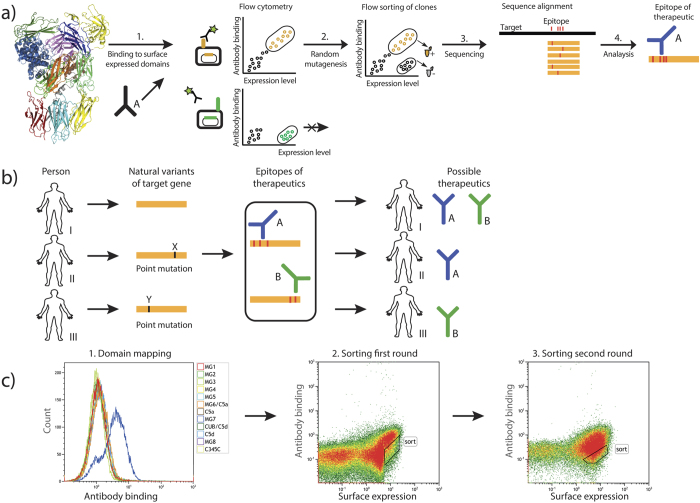
Generation and sorting of the staphylococcal mutation library. (**a**) Overview of the domain-based epitope mapping approach. Domain fragments bound by the antibody when expressed on the surface of S. carnosus are identified by flow cytometry and subsequently subjected to random mutagenesis by error-prone PCR. The mutated domain library is analysed again by flow cytometry and binding escape mutants are sorted. The isolated clones are sequenced to identify mutations that impact antibody binding. (**b**) To allow for precision medicine the genome of patients is sequenced to identify possible variations of the target gene. These are compared to the known epitopes of the therapeutics in question in order to stratify patients into relevant treatment groups. (**c**) A first domain-screening step was performed to confirm binding of eculizumab to the MG7 domain expressed on S. carnosus. The overlay histogram plot for the different C5 domains shows that antibody binding was observed when eculizumab was incubated with MG7 but not with any of the other domains displayed on S. carnosus. The events shown in the histogram plot were gated for cell size and positive expression signal. Upon mutation of MG7 some clones lost the capability to bind eculizumab. They were sorted out within gate “sort” and subjected to sequence analysis after two rounds of sorting. The dot plots represent the library before the first (left) and second (right) round of sorting, respectively. The events shown were gated for cell size.

The complement factor C5 has been structurally characterized and found to consist of twelve domains^39^,^40^. Each of the twelve domains (colour coded in the Fig. 1a) was cloned and expressed using a Gram-positive bacterial surface expression system (Supplementary Fig. S1). This allowed binding analysis of the monoclonal antibody eculizumab using a fluorescently labelled secondary antibody and flow sorting of the resulting bacterial cells with the respective domains exposed on the surface (shown schematically in Fig. 1a). The binding analysis showed that the monoclonal antibody binds exclusively to the domain MG7 (residues 822–931) expressed on Staphylococci, while all other eleven domains were shown not to interact with the antibody (Fig. 1c).

### Loss of binding mutations identified from random mutagenesis library

Having identified the MG7 domain as the binding domain for eculizumab, a random mutagenesis library of this domain was constructed by error-prone PCR (as shown schematically in Fig. 1a. The size of the staphylococcal library was 1.42 × 10^4^ clones and sequencing of 96 randomly picked clones revealed a mutation frequency of 1.4 nucleotide mutations per gene (data not shown). The albumin-binding protein (ABP), expressed in fusion with the respective mutated domain before the membrane anchoring domain, was used with labelled human serum albumin (HSA) to detect surface expression of the domain. Mutations within MG7 leading to overall misfolding of the domain or introduction of stop codons were expected not to be secreted nor properly anchored to the cell surface^34^, resulting in a decrease or absence of ABP signal for these, making it possible to avoid these during flow sorting. Clones that showed a retained high HSA signal, but a reduction in eculizumab binding were sorted in two successive rounds of flow cytometric cell sorting as indicated in Fig. 1c. After cultivating this population of enriched mutants under selective conditions on an agar plate, colonies from the sorted pool were picked and subjected to sequence analysis and characterization. Out of 90 individual clones, 71 coded for unique protein sequences with the majority containing several amino acid mutations. To facilitate data analysis and in order to be able to draw unambiguous conclusions about the contribution of a residue to antibody binding, clones containing multiple amino acid mutations were excluded from the analysis. 15 clones were identified encoding for a single amino acid mutation (Supplementary Fig. S2). Mutations involving the amino acids cysteine, glycine and proline were omitted from the analysis due to the fact that such amino acid changes often result in disruption of the overall conformation^34^. One of the clones discarded due to the mutation coding for a cysteine (R885C) had earlier been reported as a mutation present in patients^24^ and was therefore included in the subsequent analysis. This resulted in the identification of 11 clones (I829K, Q854L, F855I, R885C, R885H, K887N, K887R, K887T, V896E, W917L, F918S) with a single mutation in the MG7 domain.

**Figure 2:**
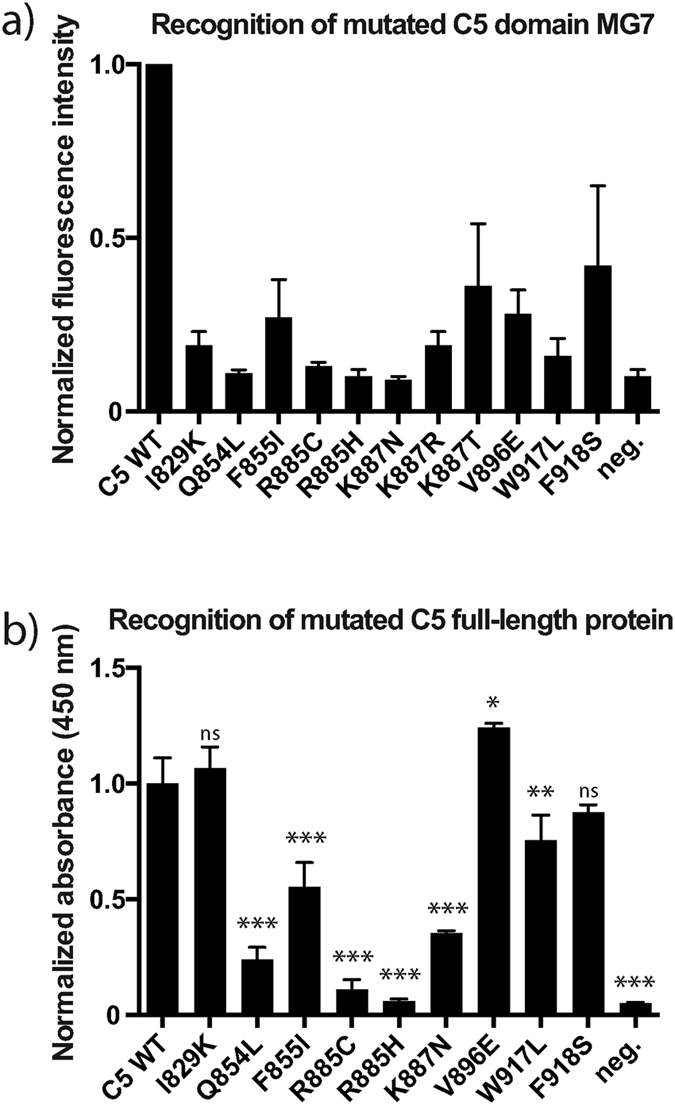
Single clone analyses of the identified mutants. (**a**) To confirm loss of binding, the identified staphylococcal mutants were analysed individually by flow cytometry. The fluorescent signal measured for WT C5 was set to 1. An unrelated protein domain (Her2 domain 1) displayed on *S. carnosus* was used as negative control. The results presented are the normalized mean and standard deviation of three independent measurements. (**b**) The diagram depicts the results of a sandwich ELISA as binding intensity of eculizumab to the full-length C5 mutants. The ELISA consists of OmCI, as capture reagent, the C5 variants, eculizumab and an anti-human, HRP-coupled detection antibody. The signal measured for non-mutant C5 (WT) was set to 1. An unrelated anit-RBM3 antibody was used instead of C5 as negative control. The results presented are the normalized mean and standard deviation of triplicates. Clones with significant loss of binding compared to WT are marked (* < 0.01; ** < 0,001; *** < 0,0001; ns=non-significant).

The 11 identified clones were analysed individually by flow cytometry for their binding to eculizumab. The analysis confirmed that they all had reduced binding to the monoclonal antibody compared to the wild type control (Fig. 2a). Some mutations, such as F918S and K887T, reduce the capability of binding to eculizumab, while others, such as Q854L, R885C/H and K887N, seem to eliminate binding completely. Interestingly, the three different mutations found at position 887 all showed reduced binding, but the degree of binding varied considerably depending on which amino acid it was changed to (asparagine, arginine or threonine). In summary, the results from the eleven clones suggest that eight different amino acids in the C5 domain are involved in the binding to the therapeutic antibody eculizumab.

### Mammalian produced full-length C5 mutants show decrease in binding to eculizumab

The wild type C5 protein and nine mutants identified above including R885C and one of the K887 mutants (K887N) were expressed and purified from transiently transfected CHO cells. A C-terminal protein C peptide tag^41^ enabled efficient affinity purification using the HPC4 monoclonal antibody. After purification, the purified C5 proteins were analysed by SDS-PAGE (Supplementary Fig. S3). The protein gel displayed two clear bands of the alpha (104 kDa) and beta chain (73 kDa) confirming a proper maturation by specific protease cleavage of pro-C5. The ability to purify both chains using the C-terminal tag of the beta-chain indicates that the two fragments are covalently interacting through native cysteine bridge.

The nine mutant C5 proteins together with the wild type C5 were analysed for binding to eculizumab by sandwich ELISA. The analysis confirmed that many of the mutants, except I829K and V896E, showed reduced binding (Fig. 2b). Although the effect for F918S is relatively low and the decrease in binding for W917L is non-significant, we decided to analyse also these mutants further. Most dramatic effect in binding to eculizumab could be attributed to the R885 mutants with almost complete loss of reactivity in the ELISA assay.

### Eculizumab cannot inhibit the haemolytic activity of the C5 mutants

**Figure 3:**
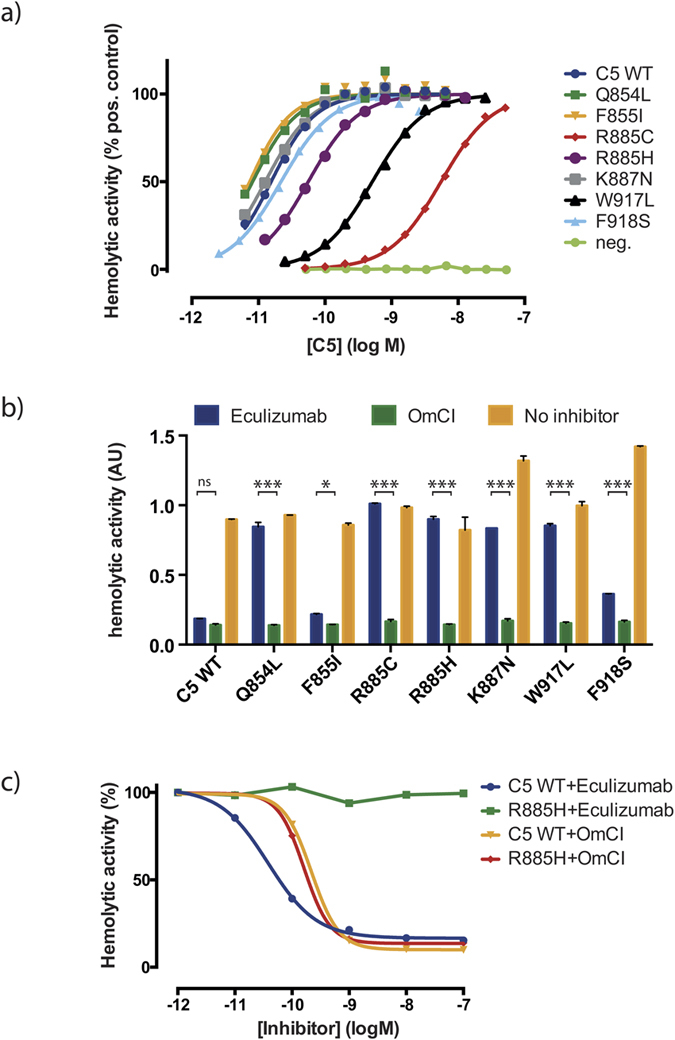
Activity and inhibition analysis of full-length C5 mutants. (**a**) Haemolytic activity was measured for mutants with reduced binding to eculizumab and for WT C5. Relative activity for each mutant is shown for different concentrations in comparison to C5 purified from plasma, whose activity at 6.4 nM was set to 100%. An unrelated anit-RBM3 antibody was used instead of C5 as negative control. (**b**) Inhibition of haemolytic activity with 3.7 nM eculizumab or 3 nM OmCI is shown for each mutant and C5 WT in comparison to uninhibited haemolysis in absorbance units (AU) at 415 nm. The results presented are the normalized mean and standard deviation of duplicates. Significant differences between eculizumab and OmCI are indicated by asterisks (ns = non-significant; * < 0.05; *** < 0.0001). (**c**) Inhibition of haemolytic activity at different concentrations of eculizumab and OmCI is shown for mutant R885H. The signal obtained without inhibitor was set to 100% haemolytic activity.

The haemolytic activity, i.e. the ability to elicit C5b-9 formation, was analysed for the recombinant C5 variants that showed reduced binding to eculizumab in ELISA to exclude that the drop in binding is due to a major structural change brought about by the mutation. The analysis shows that all mutants are able to induce C5b-9 formation in a concentration-dependent manner (Fig. 3a), although some, like R885C, do this to a lower degree than wild type C5. The lower activity of W917L could be attributed to the fact that this residue is likely to be involved in convertase binding^42^. When adding 3.7 nM eculizumab to this activity test, C5b-9 formation was inhibited in wild type C5, while mutants Q854L, R885C, R885H and W917L showed a nearly unchanged activity and F855I, K887N and F918S displayed a clearly lowered eculizumab effect (Fig. 3b and Supplementary Fig. S4). The decreased effect of eculizumab on F855I is more distinct in Supplementary Fig. S4 showing that a higher amount of eculizumab is needed to inhibit F855I compared to WT C5. When 3 nM *Ornithodorus moubata* complement inhibitor (OmCI), a tick protein^43^,^44^ that does not compete with eculizumab for binding to C5^30^, was added instead of eculizumab, C5b-9 formation was inhibited to the same degree as WT for all C5 variants (Fig. 3b,c for R885C). All analysed mutants showed a significantly reduced haemolysis when incubated with OmCI compared to eculizumab (Fig. 3b).

### The six identified residues co-locate with the epitope regions identified by crystallography

**Figure 4:**
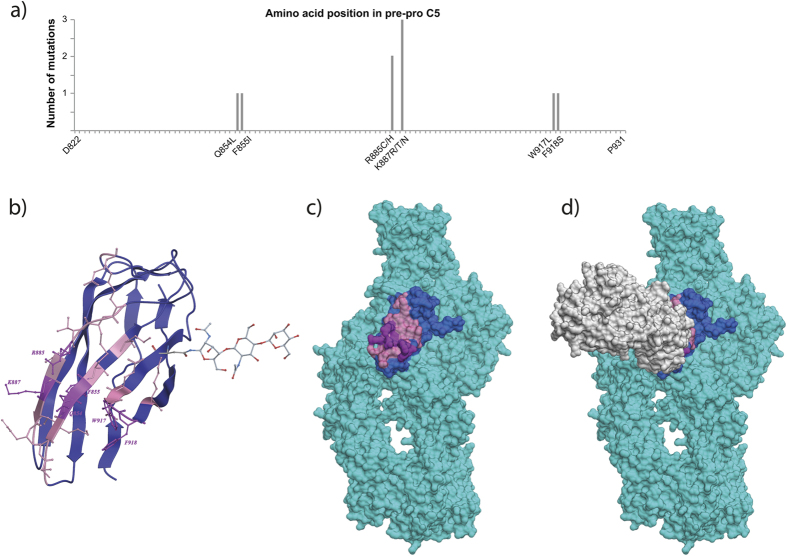
Visualization of the identified mutations on protein sequence and structure. (**a**) Illustration of the six amino acid residues identified to be involved in eculizumab binding on the MG7 amino acid sequence. The identified residues are scattered over the MG7 sequence. (**b**) Visualization of the six identified residues (in magenta) on the protein structure of domain MG7 (dark blue) (PDB5I5K). Residues within 5 Å distance from eculizumab as identified by Schatz-Jakobsen *et al.*^11^ are highlighted in rose. The six residues identified here, though on different strands, localize to the β-sheet and co-localize with the contact regions described^11^. (**c**) Visualization of the mutation sites on the structure of complete C5 (PDB5I5K). The mutated residues are highlighted in magenta, the MG7 domain in blue and residues within 5 Å distance from eculizumab as identified^11^ are highlighted in rose. The six residues (magenta) form a continuous area on the surface of C5 that is accessible to an antibody. (**d**) Illustration of the eculizumab Fab-fragment (white) bound to C5 (cyan) with the epitope highlighted as before in rose and magenta. The six residues identified in this work (magenta) locate exactly within the binding site of the antibody reported^11^.

The combined results from the binding and haemolysis experiments suggest that six residues are involved in binding to eculizumab, *i.e*. Q854, F855, R885, K887, W917 and F918. These residues are widely spread over the MG7 protein sequence (Fig. 4a) and therefore a structural analysis was done using the published co-crystal structure of C5 and an eculizumab Fab (PDB5I5K)^11^. The analysis showed that all six residues are brought together into a single site on the β-sheet in the folded C5 MG7 domain (Fig. 4b). All six residues fall into the three contact regions (residues 851–858, 882–888 and 915–920) identified by Schatz-Jakobsen *et al.*^11^ based on a less than 5 Å-distance to the Fab fragment. Further analysis revealed that, except for F855I, all five residues are more than 25% surface exposed (Fig. 4c) rendering them accessible for the antibody (Fig. 4d).

### Naturally occurring germ line mutations of C5 located in epitope

To identify possible germ line mutations in the C5 gene, three different databases (NCBI dbSNP, 1000genomes and ExAC) were searched for missense mutations in MG7. Altogether 32 naturally occurring variations in the MG7 domain were found, listed in Table 1 together with the positions of the six identified epitope residues. Two of the mutations found to abolish binding of eculizumab to C5 (R885C and R885H) were found in the database query originating from a report^24^ on poor response to eculizumab in PNH patients. In addition several genetic variations were identified in close proximity to the suggested epitope and within the 5 Å region described above^11^, which could affect the binding of the drug to a lower degree.

### W917 is specific for human C5 and is critical for eculizumab binding

It has been reported that eculizumab is specific for human C5 and that the lack of reactivity of orthologous C5 proteins has prohibited use of such animal models^5^. In Supplementary Fig. S5 the protein sequence for the epitope residues are aligned in nine different species, including primates, rat and mouse to try to explain this lack of binding. While F855 is completely conserved in all nine species, only the chemical properties of the residues at positions 887 and 918 are conserved. Residues Q854 and R885 are conserved between humans and primates, while W917 is unique for humans. All other analysed animals show a serine instead of tryptophan at position 917. Interestingly, analysis of the binding properties and haemolysis inhibition of a W917S mutant (Supplementary Fig. S6) demonstrates that this mutant has significantly decreased binding activity, thus explaining why eculizumab is non-functional in these animal models.

## Discussion

This work presents six residues on the complement protein C5 that are essential for the binding and function of the therapeutic antibody eculizumab and suggest that other means of treatment should be considered for individuals carrying mutations in these positions. All 12 domains of C5 were successfully expressed on the cell surface of *S. carnosus* and domain MG7 was identified as the binding domain of the therapeutic antibody eculizumab. A deep mutational scanning of MG7 was performed to determine the amino acids involved in eculizumab binding. After excluding multiple mutations and mutations to or from cysteine, glycine and proline that are likely to disrupt the protein structure^34^, eight residues were identified, which after functional analysis were condensed to six essential residues required for binding and function of the antibody. Although these residues are localized on different strands of the MG7 domain, they cluster to a single, contiguous site in the folded protein, suggesting that the epitope of eculizumab on C5 is dependent on tertiary structure.

This hypothesis was confirmed by the C5-eculizumab Fab co-crystal structure^11^ published after submission of this article. Schatz-Jakobsen *et al.* report three contact regions on C5 within the β-sheet of MG7 comprising residues 851–858, 882–888 and 915–920. Although the resolution of the crystallographic data did not allow the clear identification of single epitope residues, the authors inferred from the existence of binding pockets on the antibody that residues W917, F918 and R885 are involved in binding. All three residues could be confirmed to be essential for antibody binding by the surface display based epitope mapping approach presented here. In addition, three other residues, Q854, F885 and K887, which locate in the first and second contact region, described by Schatz-Jakobsen *et al.*^11^, respectively, were here shown to be important for eculizumab binding. Therefore, we regard the approach presented here using independently folded protein domains as a screening platform well suited for detection of contact residues in a structural context.

Our binding assays with full-length C5 variants from mammalian sources have shown that the findings about the eculizumab epitope on domain level on the staphylococcal surface are transferable and relevant for full-length human C5. Analysis of surface exposure reveals that all residues but F855 exceed 25% surface exposure. This might account for the lower degree of haemolytic activity, i.e. a higher degree of inhibition by eculizumab, observed for this mutant in the haemolysis inhibition assay (Fig. 3b). As Schatz-Jakobsen *et al.*^11^ we identified residue W917 as an important contact residue in this work. Our mutation studies confirmed the absence of eculizumab binding to a W917S C5 mutant. Sequence comparison showed that tryptophan at this position is specific for human C5. Considering that chimpanzee C5 only differs in two amino acids within the MG7 domain from human C5, W917 being one of them, these findings explain the species specificity observed for eculizumab, which is not able to bind to C5 of any other species but human^5^. Also R885’s involvement in eculizumab binding could be confirmed in this work. The crystal structure suggests that the mutated residues (His and Cys) are too small to fit the binding pocket^11^. Schatz-Jakobsen *et al.* also hypothesised that a cysteine mutation at that position could affect the disulphide bridge between residues 856 and 883. We observed a decreased haemolytic activity for the R885C mutant which could be explained by a structural change in the MG7 domain that perturbs interaction of this domain with the convertase^42^.

The clinical relevance of R885-mutations is highlighted by a study on Japanese PNH patients^24^ exhibiting poor response to eculizumab. Considering these insights from R885 mutation on eculizumab response, the same is thought to be true for any of the other residues found in this work to be involved in eculizumab binding. Although no germline mutations are reported for these five residues today, mutations have been reported for residues in close proximity to eculizumab as determined by the crystal structure^11^. Also mutations at these positions are likely to impair the responsiveness to eculizumab. But an analysis of single mutants would be needed to verify this. As shown in the haemolysis inhibition analysis patients with a mutation at F855, K887 and F918 would need a higher dose of eculizumab to elicit the same response as with non-mutant C5, while patients carrying a mutation at Q854, R885 and W917 likely respond so poorly to eculizumab treatment that an alternative treatment should be considered. Consequently, a clearly defined epitope as presented here can prove helpful in the clinics to screen for patients’ susceptibility to eculizumab treatment and allows for a more effective treatment.

Moreover, it could be shown that the C5 complement inhibitor OmCI, which binds at a site distinct from eculizumab^30^, was, in contrast to eculizumab, able to completely prevent the haemolysis induced by the mutant C5 molecules. Considering that OmCI, also known as coversin, was earlier reported as a possible therapeutic^30^,^45^,^46^,^47^,^48^ with low immunogenicity^45^ and is currently undergoing clinical trial, and given the results presented here, OmCI could represent an alternative future treatment strategy for patients responding poorly to eculizumab due to a missense mutation in eculizumab’s epitope on C5. Taken together our work outlines a general strategy for precision medicine based on detailed epitope mapping and genetic profiling, which should be transferable to several other diseases and therapies, and which would allow for correct treatment on those who will benefit while sparing expense and side effects for non-responders.

## Materials and Methods

### Construction of the epitope mapping library and expression in *S. carnosus*

The mRNA sequence for C5 was obtained from Source BioScience (Cambridge, UK). The C5 domains according to Fredslund *et al.*^40^ were amplified by PCR and cloned into the staphylococcal display vector pSCEM2^38^ (Supplementary Fig. 1) Transformation and library construction in *S. carnosus* is achieved as described before^49^. Briefly, the display vector pSCEM2 was amplified in *E. coli* and purified prior to transformation of *S. carnosus* TM 300 cells (a kind gift from Prof. F. Götz, University of Tübingen, Tübingen, Germany). *S. carnosus* cells were transformed by electroporation using a Bio-Rad MicroPulser electroporator at 2.3 kV for 1.1 ms. Transformed cells were grown at 37 °C overnight for protein surface expression. Having identified the C5 MG7 domain as binding domain of eculizumab (produced from CHO cells at Swedish Orphan Biovitrum AB (Sobi), Stockholm, Sweden, M.M.B, Chao Su, Lotta Berghard, Göran Selén, Kerstin Larsson, Joakim Nilsson, Catharina Sterky, Carina Ekholm, Per-Olof Edlund, Charlotte Söderberg Nyhem, P.S., manuscript in preparation), the domain was amplified using a Genemorph random mutagenesis kit (Agilent Technologies, Santa Clara, CA, USA) to yield an average mutation frequency of 1.4 mutations per sequence. The vector was purified by phenol chloroform extraction and cloned into *S. carnosus* as described above.

### Antibody labelling and analysis by flow cytometry

Of an *S. carnosus* overnight culture 2 μL were taken for analysis. The cells were washed twice with phosphate buffered saline containing 0.1% pluronic (BASF, Ludwigshafen, Germany) (PBS-P) before incubation with eculizumab (8.8 nM) for 45 min at room temperature. The cells were then washed with PBS-P and incubated with Alexa 488-labeled goat-anti-human secondary antibody (2 μg/mL) and Alexa 647-labeled HSA (1:1000 dilution) for expression control on ice in the dark for 30 min. After a final washing step the cells expressing the protein domains were analysed in a Gallios flow cytometer (Beckman Coulter, Brea, CA, USA). The same protocol was also applied when analysing the identified library clones individually.

### Sorting of the random mutagenesis library

The library clones were labelled in the same way as described above. The cells were analysed and sorted in a MoFlo Astrios cell sorter (Beckman Coulter, Brea, CA, USA). The library was sorted for binding escape mutants that show a pertained high surface expression signal but a decreased antibody-binding signal. Two rounds of sorting for binding escape mutants were performed with the sorting gates indicated in Fig. 1c to reduce the risk for false positive clones. The sorted cells were incubated on agar plates and 128 clones picked for sequence analysis.

### Production of soluble C5 variants in mammalian cells

The gene for human C5 was cloned into vector pQMCF1 coding for full-length C5 protein C-terminally fused to a tobacco etch virus-protease site and a Human protein C epitope tag (HPC4). C5 mutants were derived from this plasmid by using the Q5® Site-Directed Mutagenesis Kit (New England Biolabs, Ipswich, MA, USA). Successful mutagenesis was confirmed by Sanger sequencing. Recombinant wildtype and mutant C5 was expressed in Chinese hamster ovary (CHO) EBNALT 85 cells (Icosagen, Tartu, Estonia) following the manufacturer’s guidelines for extended transient expression. After 15 days of cultivation C5 proteins were purified from the cell culture supernatant by affinity chromatography on an anti-protein C affinity matrix (Roche, Basel, Switzerland). Due to low protein concentration, some samples were concentrated using a centrifugal filter (Merck Millipore, Darmstadt, Germany). Protein concentration was determined with ImageJ (see supplementary methods) from a Coomassie-stained sodium dodecyl sulphate polyacrylamide gel electrophoresis (SDS-PAGE) (Supplementary Fig. S3). All non-concentrated protein samples had a purity of over 95% as assessed by Coomassie staining after SDS-PAGE (Supplementary Fig. S3).

### Binding analysis of full-length, soluble C5 variants

Binding of recombinant wildtype and mutant C5 was assessed by ELISA. A 96-well half area plate was coated overnight with 33.3 nM OmCI (produced at Sobi as described in Bloom *et al.*^50^). The next day, the plate was blocked with PBS containing 1% bovine serum albumin (BSA). C5 variants were added at a concentration of 63 nM in blocking buffer. After washing the plate, eculizumab was added at a concentration of 16 nM. Antibody binding was detected with help of an anti-human IgG antibody coupled to horseradish peroxidase (HRP) and Tetramethylbenzidine (TMB) substrate. The reaction was terminated by addition of 2 M sulfuric acid. Absorbance was measured at 450 nm.

### Activity analysis of full-length, soluble C5 variants

The ability of WT and mutated C5 to form C5b-9 was monitored by haemolytic activity using antibody (Rabbit anti-sheep RBC stroma, Sigma #S1389) coated sheep red blood cells. 5 × 10^6^ cells in 50 μL GVBp (0.15 mM CaCl_2_, 0.5 mM MgCl_2_, 3 mM NaN3, 138 mM NaCl, 0.1% gelatine, 1.8 mM sodium barbital and 3.1 mM barbituric acid, pH 7.3–7.4) were used and the amount of hemolysis was monitored as absorbance at 415 nm. Diluted C5 deficient human serum (1:50) was spiked with C5 (WT or mutant) at different concentrations ranging from 51.2 nM to 6.25 pM. Subsequently, the capacity to inhibit haemolytic activity by eculizumab and OmCI at 100–0.001 nM was monitored using C5 concentrations optimized to normalize haemolytic activity between C5 variants; 0.023 nM (WT), 0.013 nM (Q854L), 0.011 nM (F855I), 8.9 nM (R885C), 0.089 nM (R885H), 0.046 nM (K887N), 0.72 nM (W917L), 1.6 nM (W917S) and 0.089 nM (F918S).

### Data analysis

The six identified amino acid mutations were visualized on an existing 3D structure of C5 (PDB5I5K)^11^ using ICM software (Molsoft LLC, San Diego, USA). For comparison of the six identified residues with naturally occurring germ line mutations, three databases, dbSNP^51^, 1000genomes^52^ and the Exome Aggregation Consortium (ExAC) browser^53^, were searched for missense mutations occurring in the MG7 coding region (residues 822–931). Furthermore, the protein sequence of human C5 was aligned to C5 sequences of chimpanzee (*Pan troglodytes*), crab-eating macaque (*Macaca fascicularis*), rhesus macaque (*Macaca mulatta*), cat (*Felis catus*), wild boar (*Sus scrofa*), cow (*Bos taurus*), rat (*Rattus norvegicus*) and mouse (*Mus musculus*). The amino acids at the six identified positions were analysed for species conservation.

### Statistical analysis

The mean of duplicates or triplicates is depicted in all column charts. Error bars indicate standard deviation. Statistical significance was calculated using GraphPad Prism Software. One-way Analysis of variance (ANOVA) with Dunnett’s multiple comparison test and two-way ANOVA with Tukey multiple comparison test was applied for ELISA results and haemolytic activity testing, respectively.

## Additional Information

**How to cite this article**: Volk, A.-L. *et al.* Stratification of responders towards Eculizumab using a structural epitope mapping strategy. *Sci. Rep.* **6**, 31365; doi: 10.1038/srep31365 (2016).

## Supplementary Material

Supplementary Information

## Figures and Tables

**Table 1:** Reported naturally occurring point mutations within the C5 MG7 domain.

C5 residue	Critical for binding	Surface-exposed residues within 5 Å from the eculizumab Fab	Genome variation
D822			Y
V823			I
I829			T
V845			L
S851		Yes	T/Y/F
Q854	Yes	Yes	
F855	Yes		
M859			I
S860			F
V862			A/L
G864			E
T867			P
S868			L/stop
E869		Yes	K
S870			T
S871			A
I873			V
D874			N
H875			R
S881			F
C883		Yes	S
V884		Yes	L
R885	Yes	Yes	C/H/S
K887	Yes	Yes	
S892			F
V896			L
F898			Y
I905			T
L907			F
H908			Y
N909			S
L914			P
W917	Yes	Yes	
F918	Yes	Yes	
E921			G
V924			G
R928			Q
