# Supplementary material for: Stratification of responders towards eculizumab using a structural epitope mapping strategy
Source: Sci Rep. 2016 Aug 11;6:31365. doi: 10.1038/srep31365 (PMC4980765; doi:10.1038/srep31365)
Supplement: Supplementary Information [file srep31365-s1.pdf]

Supplementary Information to

Stratification of responders towards eculizumab using a structural epitope mapping strategy

Anna-Luisa Volk[1,2], Francis Jingxin Hu[1,2], Magnus M. Berglund[3], Erik Nordling[3], Patrik Strömberg[3], Mathias Uhlén[1,2,4,5] and Johan Rockberg[1]

Supplementary methods

Concentration determination with ImageJ

Only the 73 kb C5 ß-chain-bands were regarded for densitometry-based concentration determination. In ImageJ, a horizontal box was placed over all samples and the albumin bands of the marker. This procedure was repeated once and the mean intensity of both analyses was used for concentration calculation. The concentrations were calculated based on the known protein concentration of the 66 kb-band of the two marker lanes. As a further control step, densitometry-based concentrations for the unconcentrated samples were compared to concentrations determined with absorbance at 280 nm which were in good agreement.

Supplementary figures

a)

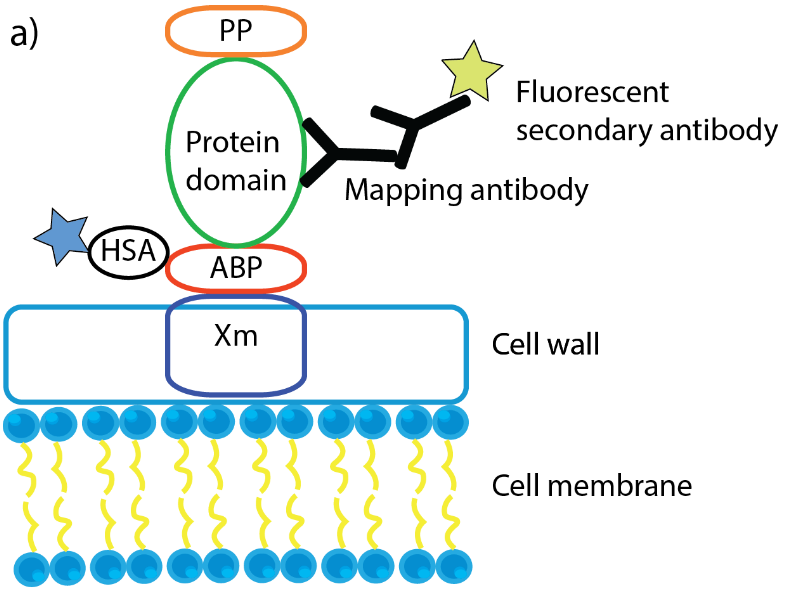

b)

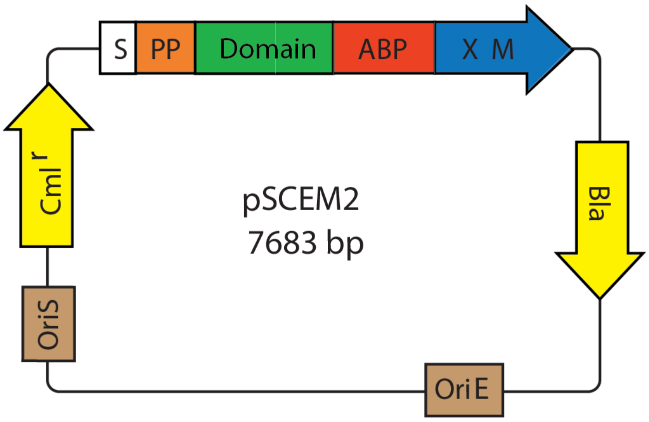

Supplementary figure S1

Schematic representation of the staphylococcal surface expression construct and the corresponding vector. **(a)** The protein domain is expressed on the cell surface together with a propeptide (PP) for efficient translocation, albumin-binding protein (ABP) for normalization of expression via human serum albumin (HSA) binding and the XM-region for cell wall anchoring. **(b)** The expression cassette encoding for signal peptide (S), propeptide (PP), the protein domain, albumin binding protein (ABP) and the cell wall anchoring region XM is encoded on vector pSCEM2 with origins of replication both in *E. coli* (OriE) and *S. carnosus* (OriS) as well as corresponding antibiotic selection markers (Cml^r, Bla)

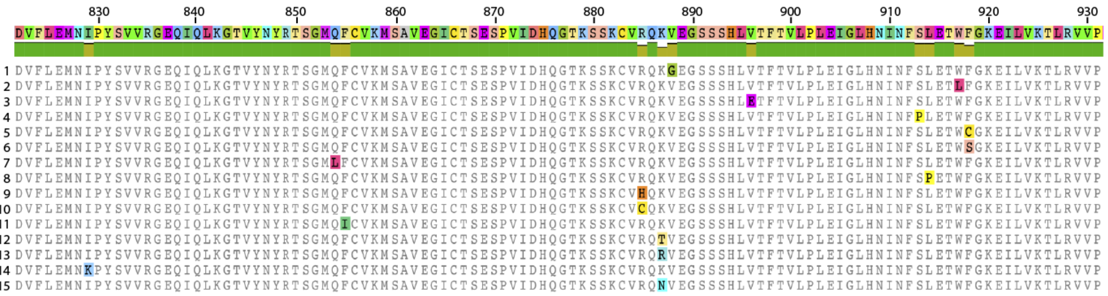

Supplementary figure S2

Protein sequence alignment of the MG7 region of the 15 single mutation clones. Among the unique sequences 15 clones, clones were identified the coded for a single mutation. The MG7 protein sequences of these 15 clones were aligned and discrepancies from the wildtype sequence are highlighted with a specific colour for every amino acid.

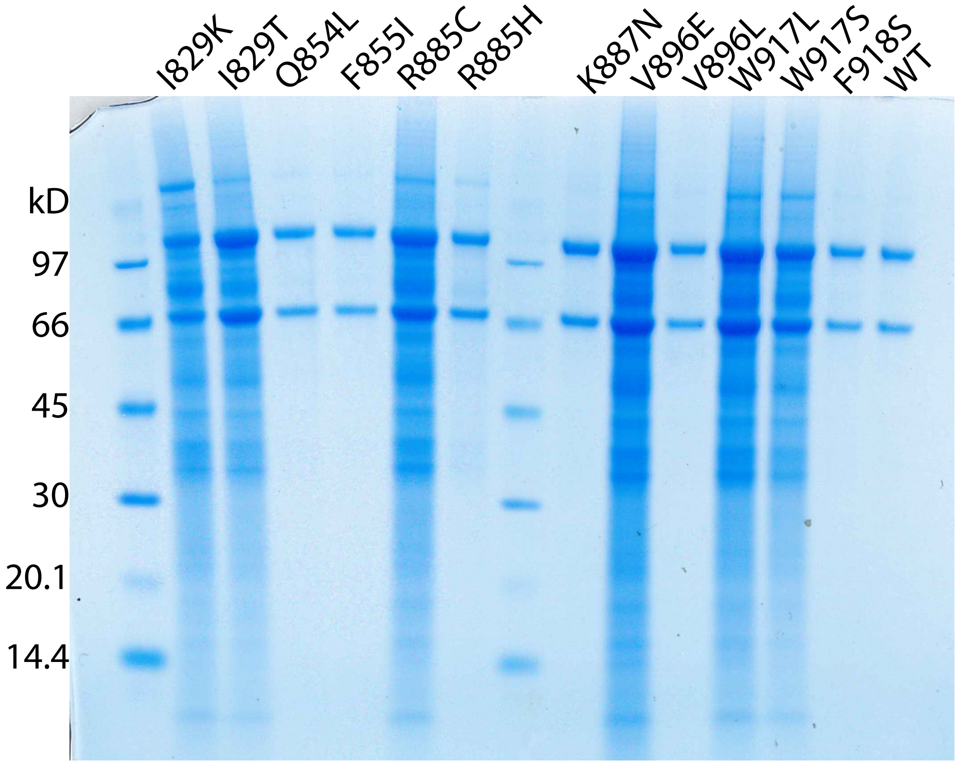

Supplementary figure S3

SDS-PAGE of purified and concentrated C5 variants. 2 µL of each C5 variant was separated on a reducing SDS-PAGE. The bands for C5's alpha (104 kD) and beta (73 kD) chain are clearly visible while barely any contaminations can be detected in the non-concentrated samples. Upon concentration of samples I829K, I829T, R885C, V896E, W917L and W917S other protein bands become visible.

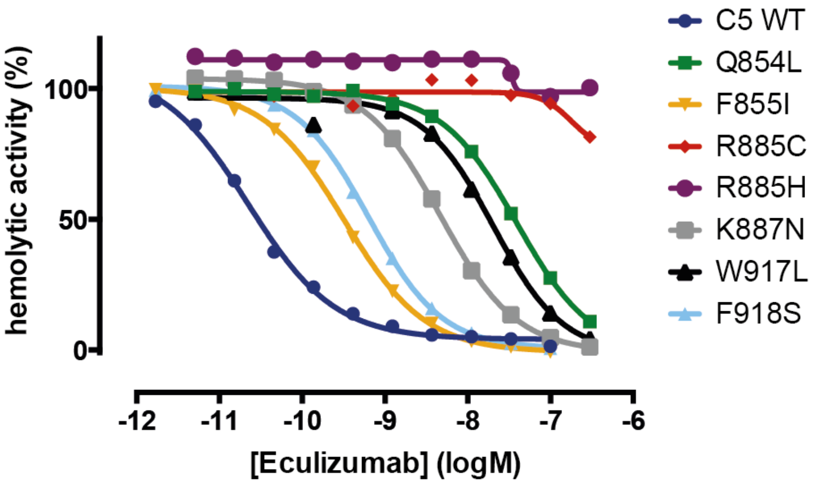

Supplementary figure S4

Analysis of inhibition of haemolytic activity of seven full-length C5 mutants. Haemolytic activity was measured for each mutant and WT C5 at increasing concentrations of Eculizumab. Relative activity at different inhibitor concentrations was calculated for each mutant in comparison to uninhibited haemolysis.

Uniprot accession ID	Species	pos 854	pos 855	pos 885	pos 887	pos 917	pos 918
CO5_HUMAN	Homo sapiens	Q	F	R	K	W	F
H2QXT1_PANTR	Pan troglodytes	Q	F	R	K	S	F
G7PRJ3_MACFA	Macaca fascicularis	Q	F	R	K	S	F
F7GHV1_MACMU	Macaca mulatta	Q	F	R	K	S	F
M3VV58_FELCA	Felis catus	R	F	S	R	S	L
Q9VPV1_PIG	Sus scrofa	L	F	P	K	S	L
F1MY85_BOVIN	Bos taurus	Q	F	L	K	S	V
A0A096P6L9_RAT	Rattus norvegicus	M	F	R	R	S	F
CO5_MOUSE	Mus musculus	K	F	F	R	S	F
		Q854 conserved in primates	F855 conserved	R885 conserved in primates	K887 conserved property	W917 human specific	F918 property conserved

Supplementary figure S5

Comparison of identified residues with C5 sequences of other species. The six identified residues on human C5 were compared to amino acids found at those positions in chimpanzee, crab-eating macaque, rhesus macaque, cat, wild boar, cow, rat and mouse. A tryptophan a position 917 was found to be specific for human C5.

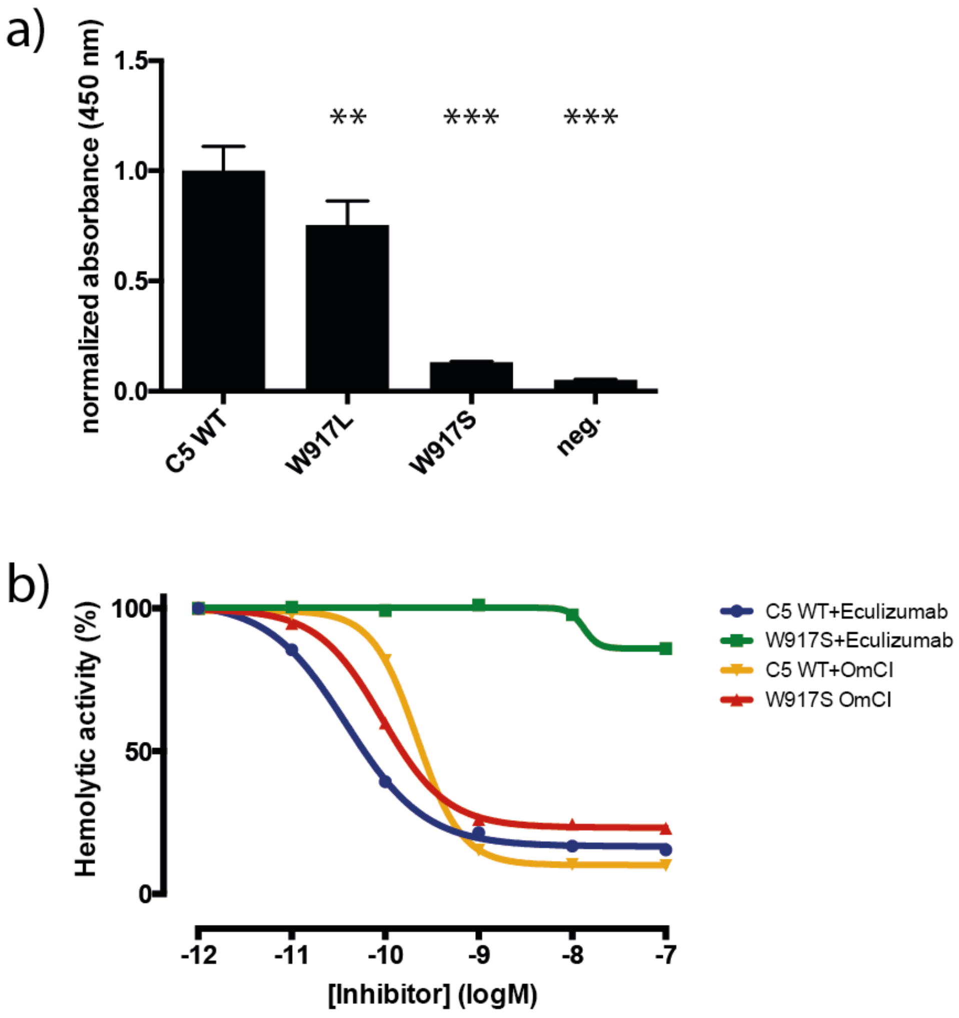

Supplementary figure S6

Binding and haemolysis inhibition analysis of W917S mutant. (a) Binding intensity to eculizumab was determined by sandwich ELISA using, sequentially, OmCI as capture reagent, different C5 variants, eculizumab and an anti-human HRP-coupled detection antibody. An irrelevant anti-RMB3 antibody was used instead of C5 as negative control. The signal measured for non-mutant C5 was set to 1. The results presented are the normalized mean and standard deviation of triplicates. W917S shows significantly reduced binding to Eculizumab in ELISA (**$p<0{,}001$; ***$p<0.0001$). **(b)** Inhibition of haemolytic activity with increasing concentrations of Eculizumab and OmCI is shown. Relative activity at different inhibitor concentrations is shown for W917S mutant in comparison to non-mutant C5. While the W917S mutant is basically unaffected by Eculizumab, OmCI inhibits this mutant comparable to WT C5.
